# Glutamine and glutamate supplementation raise milk glutamine concentrations in lactating gilts

**DOI:** 10.1186/2049-1891-3-2

**Published:** 2012-02-28

**Authors:** Helena Emilia CCC Manso, Helio C Manso Filho, Luiz E de Carvalho, Marianne Kutschenko, Eduardo T Nogueira, Malcolm Watford

**Affiliations:** 1 Department of Animal Sciences, Rural Federal University of Pernambuco, Recife, PE, 52171-900, Brazil; 2 Departament of Animal Sciences, Federal University of Ceará, Fortaleza, CE, 60020-181, Brazil; 3 Ajinomoto do Brazil, São Paulo, SP, 04015-001, Brazil; 4 Department of Nutritional Sciences, Rutgers University, New Brunswick, NJ 08901, USA

**Keywords:** glutamate, glutamine, lactation, milk, pig, skeletal muscle

## Abstract

Glutamine is the most abundant amino acid in milk, and lactation is associated with increased glutamine utilization both for milk synthesis and as a fuel for the enlarged small intestine. A number of recent studies have indicated that lactation is accompanied by a mild catabolic state in which skeletal muscle proteins are degraded to provide amino acids that are used to synthesize additional glutamine. In this study we tested the hypothesis that supplemental L-glutamine or the commercially available glutamine supplement Aminogut (2.5% by weight mixed into daily feed) provided to gilts from 30 days prior to parturition until 21 days post-parturition would prevent a decrease in skeletal muscle glutamine while increasing the glutamine content of the milk. Muscle glutamine content decreased (*P *< 0.05) in control animals during lactation but this was prevented by supplementation with either L-glutamine or Aminogut. In this study, neither lactation nor supplementation had any effect on plasma glutamine or glutamate content. Free glutamine, and the total glutamine plus glutamate concentrations in milk from the control and the Aminogut group rose (*P *< 0.05) during the first 7 days of lactation, with milk concentrations in the L-glutamine supplemented group showing a similar trend (*P *= 0.053). Milk glutamate remained constant between day 7 and 21 of lactation in the control and L-glutamine supplemented groups, but by day 21 of lactation the free glutamine, glutamate, and glutamine plus glutamate concentrations in milk from Aminogut-treated gilts were higher than those of control gilts. Thus dietary glutamine supplementation can alleviate the fall in intramuscular glutamine content during lactation in gilts, and may alleviate some of the catabolic effects of lactation. Furthermore, the increased milk glutamine content in the supplemented gilts may provide optimum nutrition for piglet development.

## Introduction

Glutamine is the most abundant free -amino acid in the body of most mammals and the majority of stored glutamine is concentrated in skeletal muscle [1,2]. Glutamine and glutamate comprise between 5 and 15% of the amino acid content of most foods and commercial feedstuffs, but since these amino acids are effectively metabolized by the epithelial cells of the small intestine there is little net absorption at normal dietary intake levels [1]. Thus, the large glutamine pool in the body arises from *de novo *synthesis in the skeletal muscle through the action of glutamine synthetase. Catabolic stress increases the need for glutamine by a number of tissues. This demand is met by increased glutamine release from skeletal muscle. Initially, release of glutamine from the existing intramuscular pool results in a drop in the intracellular glutamine concentration. However, continued release of glutamine requires increased synthesis. An increase in net proteolysis within the muscle cells provides the amino acid substrates for glutamine synthesis [3,4]. We recently determined that, in the horse, lactation represents a mild catabolic state accompanied by a loss of lean body mass and a decrease in muscle glutamine content [5]. Additional evidence supports a similar conclusion for a number of other species, including the pig [6-11].

It is well established that during lactation there is increased glutamine utilization since glutamine is the most abundant amino acid in the milk of most species [12-17]. Additionally, enlargement of the intestines during pregnancy and lactation also increases the glutamine requirements of the intestinal epithelial cells [18]. The source of the substrates for increased glutamine synthesis to meet these demands has not been definitively identified, but it is generally thought that amino acids obtained from increased dietary protein intake would suffice [18]. However, our work with the horse suggests that, in addition to dietary amino acids, muscle proteolysis also provides some of the substrates for glutamine synthesis during lactation [5]. Therefore, we hypothesized that supplemental dietary glutamine (and/or glutamate) could provide the extra glutamine required for milk production, thereby limiting the need to utilize endogenous proteins and aiding in the maintenance of lean body mass. In this study we tested this hypothesis in gilts and, in addition, we investigated whether dietary glutamine supplementation can raise milk glutamine concentrations.

## Materials and methods

### Animals

Forty-five gilts (Topigs, Dalland Genetics, Campinas, SP, Brazil) were selected 30 days prior to farrowing (last third of gestation) and were housed at Tangueira Farm, Maranguape, Fortaleza, Ceará, Brazil. From 30 days prior to farrowing until parturition all gilts were fed 2.0 kg per day of a commercially available gestation feed divided into two meals (morning and evening). After farrowing all gilts were fed a lactation feed, and their feed allowance was gradually increased until it reached approximately 4.0 kg daily by day seven of lactation. Both feeds were designed to provide adequate nutrition for pigs in the relevant phase of the reproductive cycle (Table 1). Feed was provided in individual automatic feeders, and water was available *ad libitum*.

**Table 1:** The nutritional composition of the commercial feeds fed to the pregnant and lactating gilts.

Ingredients	Gestation feed	Lactation feed
Main ingredients, kg		

Corn (grain)	534.0	574.0

Wheat middlings	320.0	40.0

Soybean meal	81.0	132.0

Extruded full-fat soybeans	--------	186.0

Meat meal	50.0	12.0

Sugar	-------	20.0

Salt	5.0	5.0

Limestone 38%	4.0	9.0

Dicalcium phosphate	--------	16.0

Microingredients		

Trace mineral premix	0.5	0.5

Chromium	1.0	1.0

DL-Methionine 98%	--------	0.198

L-Lysine 80%	0.381	0.356

Penicillin 98%	---------	0.2

Breeding premix	4.0	4.0

Total, kg	1,000.0	1,000.0

Nutrients		

Crude protein, %	15.48	19.0

Crude fiber, %	4.66	3.65

Ashes, %	5.28	6.14

Calcium, %	0.8	0.96

Total phosphorus, %	0.72	0.71

Metabolizable energy, kcal/kg	2,900.5	3,348.3

Source: Nutron Alimentos LTDA (Brazil)

From 30 days before until 21 days after farrowing the gilts were divided into three groups, which received dietary supplementation as follows: control group, no supplementation; glutamine group, L-glutamine supplementation (2.5% by weight mixed into the daily ration); Aminogut group, Aminogut supplementation (2.5% by weight mixed into the daily feed). Aminogut is a commercially available dietary supplement produced by Ajinomoto do Brazil (São Paulo, Brazil) that contains both free glutamine (min 10%) and glutamic acid (min 10%). On the day of farrowing all litters were reduced to 10 or 11 piglets; however, since the work was carried out on a commercial farm it was only possible to determine the body weight of the piglets on the day of birth and at weaning. All animal work was approved by CEUA - Ethical Committee of Animal Utilization/UFRPE (Federal Rural University of Pernambuco).

### Samples

Blood and muscle samples were taken 30 days prior to parturition, at parturition and on days 7 and 21 of lactation; milk samples were taken at parturition and on days 7 and 21 of lactation. All sampling was done 2 to 3 hours after the morning feed. Blood samples were drawn from an auricular vein and placed on ice. Muscle samples were obtained by biopsy of the superficial gluteus muscle. Briefly, a small incision was made in the skin and adipose tissue over the superficial gluteus muscle to allow the introduction of the biopsy needle and the muscle was sampled at a depth of approximately 8 cm below the skin (well beyond the fat layer). After collection, muscle samples were stored at -80°C until analysis. Only the control animals underwent the initial muscle biopsy at 30 days before parturition. To facilitate milk collection the gilts received an injection of oxytocin (0.5 mL, i.v.; Prolacton 1:10000, Tortuga, Brazil). Milk was collected from the abdominal teats and immediately placed on ice.

Blood and milk samples were deproteinized by the addition of an equal volume of perchloric acid (10% w/v), and muscle samples (100 to 200 mg) were homogenized in 4 to 5 volumes of perchloric acid (10% w/v). The acid extracts were then centrifuged (5,000 g for 15 minutes) to remove proteins and the supernatants neutralized with potassium hydroxide and stored at -80°C until analysis.

### Glutamine and glutamate determination

Glutamine was hydrolyzed to glutamate by glutaminase. Glutamate levels were then determined enzymatically using glutamate dehydrogenase and the reduction of NAD^+ ^[19].

### Statistical analysis

Gilts that were unable to maintain 10 to 11 piglets were excluded from the study. The number of gilts that completed the study and were included in the statistical analysis were 6, 5 and 8 in the control, glutamine, and Aminogut groups, respectively. All results were analyzed by ANOVA using SAS Statistical Package version 9.2 (SAS Institute, Cary, NC). Dunnett's test was used to compare body weight, back fat thickness, and glutamine/glutamate in blood and muscle during lactation to the pre-farrowing value. Tukey's post hoc testing was used to compare differences between individual groups (time and treatment) in milk concentrations. Differences were considered statistically significant if *P *< 0.05.

## Results

### Animals

All gilts lost weight during the 21 days post-farrowing (Table 2), but there were no significant between-group differences in weight at lactation day 21 expressed as a percentage of parturition day weight. Similarly, all gilts showed a decrease in back fat thickness over the first 21 days of lactation, but there were no between-group differences in the thickness remaining at day 21 post-farrowing as a percentage of the thickness at 30 days prior to farrowing. Litter size and piglet weight at birth and weaning did not significantly differ between groups (Table 3).

**Table 2:** Body weight and back fat thickness in gilts

	Control (n = 6)	Glutamine (n = 5)	Aminogut (n = 8)
Body weight, kg			

30d pre-farrowing	166.6 ± 8.1	185.1 ± 9.1	175.2 ± 6.5

0d (parturition)	182.7 ± 6.2	185.6 ± 9.5	174.5 ± 9.8

7d post-farrowing	176.3 ± 5.2	181.7 ± 9.4	162.0 ± 5.2

21d post-farrowing	165.0 ± 5.2	157.5 ± 9.7	147.8 ± 7.4*

Backfat, cm			

30d pre-farrowing	17.2 ± 0.9	18.4 ± 1.8	15.3 ± 0.4

0d (parturition)	16.5 ± 0.5	23.6 ± 2.9	16.1 ± 0.8

7d post-farrowing	15.3 ± 1.6	19.8 ± 2.5	14.0 ± 0.6

21d post-farrowing	13.7 ± 1.9	13.4 ± 1.9*	11.3 ± 0.4*

Results are expressed as means ± SEM

* indicates significantly different from the 30d pre-farrowing value for that group (*P *< 0.05)

**Table 3:** Litter size and piglet body weight at birth and weaning (21 days)

	Control (n = 6)	Glutamine (n = 5)	Aminogut (n = 8)
Body weight, g			

Birth	1.59 ± 0.04	1.39 ± 0.17	1.27 ± 0.07

Weaning	5.92 ± 0.27	6.27 ± 0.63	5.67 ± 0.29

Number of piglets			

Birth	65	55	93

Weaning	64	54	84

Results are expressed as means ± SEM. There were no significant differences between groups.

### Muscle glutamine and glutamate

**Figure 1:**
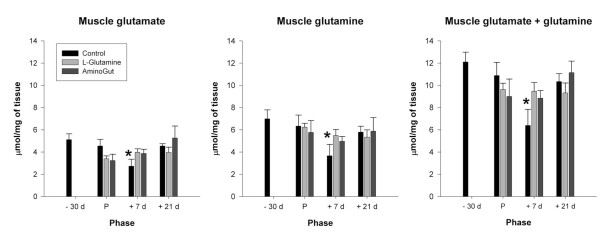
**Muscle glutamate, glutamine, and glutamate plus glutamine concentration in gilts**. Results are expressed as means ± SEM for 6 control, 5 glutamine and 8 Aminogut gilts. Phase refers to -30d (30 days pre-farrowing), P (parturition), +7d (7 days post-farrowing), +21d (21 days post-farrowing). *indicates significantly different from the pre-farrowing value for that group.

Muscle free glutamine, glutamate, and glutamine plus glutamate concentrations in the control group at 7 days after farrowing were significantly lower than at 30 days prior to parturition (Figure 1). Supplemented animals did not differ in muscle glutamine or glutamate content when these values were compared with the values obtained from the control group 30 days prior to parturition.

### Blood glutamine and glutamate

**Figure 2:**
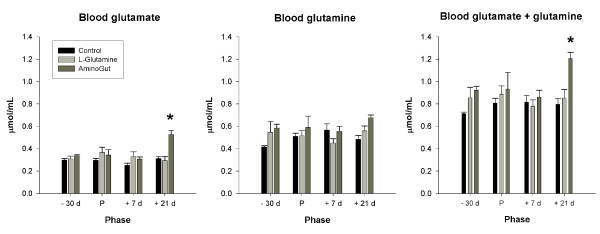
**Blood glutamate, glutamine, and glutamate plus glutamine concentration in gilts**. Results are expressed as means ± SEM for 6 control, 5 glutamine and 8 Aminogut gilts. Phase refers to -30d (30 days pre-farrowing), P (parturition), +7d (7 days post-farrowing), +21d (21 days post-farrowing). *indicates significantly different from the pre-farrowing value for that group.

Blood glutamate and glutamate plus glutamine concentrations at 21 days post-farrowing were higher in the Aminogut group than in the control and glutamine groups. These levels also differed significantly from the pre-farrowing levels in the Aminogut group only (Figure 2).

### Milk glutamine and glutamate

**Figure 3:**
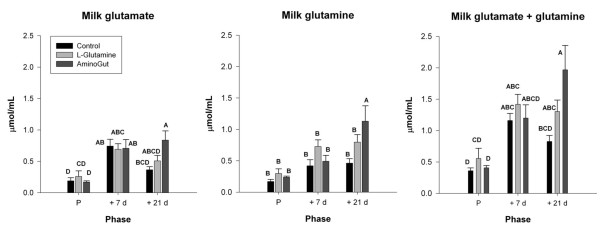
**Milk glutamate, glutamine, and glutamate plus glutamine concentration in gilts**. Results are expressed as means ± SEM for 6 control, 5 glutamine and 8 Aminogut gilts. Phase refers to P (parturition), +7d (7 days post-farrowing), +21d (21 days post-farrowing). Values with different superscripts are significantly different (*P *< 0.05).

Milk free glutamate content in the control and Aminogut groups rose between parturition and day 7 post-farrowing (Figure 3). In the glutamine supplemented group, milk glutamate content showed a trend (*P *= 0.053) to be higher at 7 days post farrowing than at parturition. Between days 7 and 21 of lactation further changes in milk glutamate content did not reach significance in any group, but by day 21 of lactation the milk glutamate content of the Aminogut group was significantly higher than that of the control group. Milk glutamine content was relatively constant in the control and glutamine groups, but by day 21 post-farrowing the milk glutamine content of the Aminogut group was higher than in the other groups. The total content of glutamine plus glutamate in the milk rose between parturition and day 7 post-farrowing in the control group, and showed a trend towards increase (*P *= 0.078) in the Aminogut group. By 21 days post-farrowing total glutamine plus glutamate was higher in both the Aminogut and glutamine groups relative to their values on the day of parturition, and levels in the Aminogut group were much higher than in the control group at 21 days post-farrowing. At day 21 post-farrowing the glutamine plus glutamate concentration in the Aminogut-supplemented animals was 240% of the concentration in milk from control animals and 175% of the concentration in milk from the glutamine-treated group.

## Discussion

Traditionally glutamine is not considered an essential amino acid. The body is able to synthesize considerable quantities of glutamine (and glutamate), and in healthy adult mammals there is no evidence of any glutamine shortage [1,2]. However, during times of severe trauma or infection (hypercatabolic states) there is an increase in the glutamine requirement of a number of tissues, including the immune cells, the liver and the kidneys. Increased glutamine is then supplied by *de novo *synthesis from amino acids derived from the breakdown of skeletal muscle protein and an increase in the activity of skeletal muscle glutamine synthetase. Despite this increase in glutamine synthesis and release by the muscle, there is considerable evidence that under conditions of severe stress endogenous glutamine production is insufficient to meet the body's needs. A number of clinical trials have shown benefits of both enteral and parenteral glutamine supplementation, with successful trials involving large (up to and above 50 g glutamine per day per person) amounts of glutamine supplementation [3,4]. There is little evidence, however, of any beneficial effects of glutamine supplementation in healthy individuals [20].

In our work with lactating horses we discovered that lactation was accompanied by a decrease in glutamine pools and a loss of lean body mass [5]. This led us to conclude that lactation was a mildly catabolic state in this specie, and additional studies have reported that lactation represents a mild, transient catabolic state in a number of other species including the pig [6-11]. While it is well recognized that glutamine needs are greatly increased during lactation, both to provide milk glutamine and to fuel the enlarged small intestine, it has usually been supposed that additional glutamine to meet this demand would simply be synthesized from the extra dietary amino acids consumed during lactation [18]. Our findings in the horse suggest that dietary amino acids are insufficient to meet this increased demand, and that the mare draws additional substrates from her own lean mass to provide the glutamine required by other tissues. In our current work, lower glutamine/glutamate concentrations in skeletal muscle early (day 7) in lactation in gilts receiving the control diet also indicate that a mild catabolic state is present in early lactation. That supplemental glutamine/glutamate prevents such changes during lactation indicates that perhaps lean body mass may be maintained during lactation with appropriate dietary intervention.

In addition to sparing the lean body mass of the sow, supplemental glutamine could also result in an increase in milk glutamine. Since glutamine is readily hydrolyzed to glutamate and is thus relatively labile during storage, consideration of glutamine plus glutamate together in stored samples might best reflect changes in the glutamine pool *in vivo*. In addition, the combined concentrations of glutamine and glutamate are additionally relevant since the pig intestine metabolizes both dietary glutamine and glutamate similarly.

The high glutamine content in the milk of most species is likely due to the high glutamine needs associated with rapid growth and cell division in neonatal tissues, particularly in the neonatal small intestine and gut-associated lymphoid tissue (GALT). It is well established that the neonatal gut is particularly sensitive to stress, and that abrupt early weaning is often associated with negative growth and pathological outcomes that are clearly related to intestinal dysfunction. A number of studies over the past 15 years have shown that glutamine and/or glutamate supplementation can be beneficial to piglet gut health and weight gain [15,21-27]. Most of these studies began at weaning, but at least one addressed the question of supplemental glutamine during the suckling period. Wu and colleagues [15] gave suckling piglets glutamine (3.42 mmol/kg) twice daily by oral gavage and found improvements in intestinal function and growth performance. Such findings strongly support the idea that maternal milk does not usually contain optimal amounts of glutamine (and/or glutamate) and that provision of exogenous glutamine is beneficial to suckling animals. For most species it is not practical to supply supplemental amino acids by gavage, but our results indicate that supplementation of the sow may offer an alternative means of increasing glutamine delivery to the piglet. The concentration of glutamine/glutamate in milk from gilts receiving supplemental glutamine and glutamate (particularly in the Aminogut group) was double that of the control animals. Thus, piglets suckling from supplemented mothers received more dietary glutamine and glutamate, and any potential associated benefits would also likely be received. In this study, supplemental glutamine or Aminogut did not change litter size or piglet weight at birth or weaning. A similar study that supplemented the diet of lactating sows with 2.5% glutamine also showed higher milk glutamine concentrations at days 7 and 21 of lactation with no differences in litter parameters except for some small effects on piglet intestinal anatomy [22]. Our study was conducted on a commercial pig farm, therefore it was not possible to sample the piglets or to follow them in the immediate post-weaning period. Thus further research is needed to quantify the benefits of providing extra glutamine to the piglets.

During lactation, increased glutamine utilization requires an increase in endogenous glutamine production. In skeletal muscle, the branched chain amino acids that are the substrates for increased glutamine production are derived from dietary protein or from intracellular proteolysis. Our work, together with numerous other studies, indicates that lactation is accompanied by a loss of lean body mass that is, in part, related to an increase in muscle glutamine synthesis. The finding that skeletal muscle glutamine content is maintained in animals receiving glutamine/glutamate supplementation indicates that suitable dietary interventions could ameliorate some of the negative effects of lactation in gilts. Furthermore, the increased glutamine and glutamate in the milk of the supplemented gilts may improve the growth and intestinal health of their piglets throughout the suckling, weaning and post-weaning periods.

## Competing interests

The authors declare that they have no competing interests.

## Authors' contributions

HECCCM participated in the design of the study, carried out the animal work, the biochemical analyses, statistical analyses and wrote the first draft of the manuscript. HCMF participated in the design of the study and the statistical analyses. LEC, MK, ETN and MW participated in design of the study. All authors contributed to the writing of the final versions of the manuscript and have read and approved the final manuscript.
